# Notch signaling plays a crucial role in cancer stem-like cells maintaining stemness and mediating chemotaxis in renal cell carcinoma

**DOI:** 10.1186/s13046-017-0507-3

**Published:** 2017-03-09

**Authors:** Wei Xiao, Zhiyong Gao, Yixing Duan, Wuxiong Yuan, Yang Ke

**Affiliations:** Department of Urology, Hunan Provincial People’s Hospital, JiefangWest Road 61, Changsha, Hunan China

**Keywords:** Renal cell carcinoma, Cancer stem cells, Notch pathway, Stemness sustaining, Chemotaxis, SDF-1/CXCR4

## Abstract

**Background:**

Cancer stem cells (CSCs) are correlated with the initiation, chemoresistance and relapse of tumors. Notch pathway has been reported to function in CSCs maintenance, but whether it is involved in renal cell carcinoma (RCC) CSCs maintaining stemness remain unclear. This study aims to explore the effect of Notch pathway on stemness of CSCs in RCC and the underlying mechanisms.

**Methods:**

The CD133^+^/CD24^+^ cells were isolated from RCC ACHN and Caki-1 cell line using Magnetic-activated cell sorting and identified by Flow cytometry analysis. RT-PCR and immunoblot analyses were used for determining the stemness maker expression. The effect of Notch pathway on function of CSCs was assessed by self-renewal ability, chemosensitivity, invasive and migratory ability tumorigenicity in vivo using soft agar colony formation assay, sphere-forming assay, MTT assay, Transwell assay.

**Results:**

Here, we found that the sorted CD133^+^/CD24^+^cells possessed elevated stemness maker CTR2, BCL-2, MDR1, OCT-4, KLF4, compared with parental cells, as well as enhanced self-renewal ability, stronger resistance to cisplatin and sorafenib, increased invasion and migration, and higher tumorigenesis in vivo, suggesting the CD133^+^/CD24^+^ cells have the stem-like characteristics of CSCs and thus identified as RCC CSCs. Then the enhanced notch1, notch2, Jagged1, Jagged2, DLL1 and DLL4 expression were detected in RCC CSCs and blockage of Notch1 or notch2 using pharmacological inhibitor MRK-003 or its endogenous inhibitor Numb resulted in loss of its stemness features: self-renewal, chemoresistance, invasive and migratory potential, and tumorigenesis in vivo. Moreover, it is confirmed that overexpression of notch1 up-regulated CXCR4 inRCC CSCs and augmented SDF-1-induced chemotaxis in RCC CSCs in vitro, which could be rescued when treatment of CXCR4 inhibitor, suggesting that notch signaling promotes the chemotaxis of RCC CSCs by SDF-1/CXCR4 axis.

**Conclusions:**

Our results provide a new mechanism of RCC CSCs maintaining stemness via notch pathway as well as a potential therapeutic target in human RCC.

## Background

Renal cell carcinoma (RCC) is the seventh most common tumor which is associated with high mortality [1]. RCC accounts for 2–3% of all malignant diseases in adults [2] The incidence of RCC is rising worldwide and is ∼ 209,000 cases/year and 102,000 deaths/year [3]. In addition, The renal cancer 5-year survival rate is stage dependent and ranges from 8 to 81% for TNM stage IV and I, respectively [4]. Up to 30% of RCC patients have metastatic spread at the initial presentation and the 5-year survival rate drops to 10% in patients with metastatic disease [5, 6]. Moreover, RCC recurs within the first 5 years in 40% of patients with an initially localized disease even after a nephrectomy [1]. Besides, renal cancer is extremely resistant to chemotherapy and radiation therapy [7]. It is gradually accepted that the initiation, chemoresistance, metastasis and recurrence of tumors are driven by a small subpopulation of cells endowed with stem-like properties called cancer stem cells (CSCs).

The CSCs have self-renewal capacity and differentiation potential, and can reconstruct the phenotypic and histologic heterogeneity of its parent tumor while transplanted in vivo [8, 9]. The CSCs have been isolated and identified in human renal cell carcinoma from solid tumor tissues and established cell lines [10–12], using Magnetic-activated cell sorting (MACS) or flow cytometry system based on CD133, CD24, CD105, ALDH1, Hoechst 33,342 and so on [13]. Although CD133 are commonly used a screening maker in various tumors [14, 15], it’s suggested that only CD133 may not be sufficient for CSC identification in RCC [16]. Here, we will for the first time isolate CD133^+^/CD24^+^ cells from RCC ACHN cell line using MACS and validate the expression of its stemness-associated makers (CTR2, BCL-2, MDR1, OCT-4, KLF4) and its stem-like characteristics including self-renewal capability, chemoresistance, metastatic potential and tumorigenicity in vivo.

Notch signaling represents a type of direct cell-cell communication that is essential for regulation of proliferation, apoptosis, and fate decisions of stem cells during embryonic development [17, 18]. In mammals, there are 5 Notch ligands (Delta-like [Dll] 1, 3, 4, and Jagged 1, 2) and 4 Notch receptors (Notch 1–4), all of which are type I transmembrane proteins. Activation of notch receptor results in NICD releasing into the nucleus, subsequently activating the related target genes. Increasing evidence suggest that notch pathway may promote the proliferation, survival, self-renewal, differentiation, angiogenesis, and migration of CSCs in several malignancies [19–21]. It is worth to pay attention to that the notch pathway may play either an oncogenic role or a suppressor in tumor development based on the special tumor cell context [22]. Although RCC CSCs have been identified, the expression profile of notch pathway in RCC CSCs and whether it involves maintaining the stemness of RCC CSCs and the potential molecular mechanisms remain unclear.

In this study, the CSCs models derived RCC ACHN and Caki-1 cell line were established and the expression pattern of notch1-3 and its ligands in RCC CSCs was identified. The effects of notch signaling on RCC CSCs maintaining stemness were investigated. Our results provided novel mechanisms of RCC CSCs maintenance controlled by notch signaling pathway.

## Methods

### Cell lines and medium

Human renal cancer cell lines ACHN and Caki-1 cells were purchased from ATCC (Manassas, VA) and maintained in Dulbecco’s modified Eagle’s medium (DMEM, GIBCO) supplemented with 10% fetal bovine serum (FBS, GIBCO) L-glutamine, sodium pyruvate, Penicillin/Streptomycin, at 37 °C, 5% CO_2_ condition. To compare the differences of stemness markers and features, the sortedCD133^+^, CD133^−^, CD133^+^/CD24^−^, CD133^+^/CD24^+^ cells or its responding parental cells were cultured in 6-well ultra-low plates (Corning, Acton, USA) containing serum-free medium DMEM/F12 (Gibco, Carlsbad, USA), supplemented with commercial hormone mix B27 (Gibco), 20 ng/ml EGF (PeproTech, Rocky Hill, USA), 10 ng/ml bFGF (PeproTech), 0.4% bovine serum albumin (Gibco), 4 mg/ml insulin (Gibco), 100 U/ml penicillin and 100 U/ml streptomycin at 37 °C. For CD133^+^/CD24^+^cells or CSCs maintaining culture, after being cultured for 6 days, the tumor spheres were collected, dissociated into single cell suspension and resuspended in fresh medium for serial subcultivation every 6 days.

### Magnetic bead cell sorting

For magnetic cell sorting, cells were labeled with CD133 microbeads human antibody (MiltenyiBiotec, Germany). Sorting was carried out with the Miltenyi Biotec MidiMACS Starting Kit according to the manufacturer’s instructions. Magnetic separation was performed up to three times to obtain a CD133^+^ populationmore than 70% pure. The sorted CD133^+^ cells were labeled with CD24 microbeads human antibody (MiltenyiBiotec, Germany), and magnetic separation was performed up to three times to obtain a CD133^+^/CD24^+^ populationmore than 95% pure. Aliquots of CD133^+^ and CD133^+^/CD24^+^ sorted cells were evaluated for purity with Flow cytometry analysis.

### Flow cytometry analysis

The cells were dissociated into single cells and labeled with PE-conjugated anti-CD24 and FITC-conjugated anti-CD133 (BD PharMingen) at 4 °C for 20 min. Concentrations of antibodies were used according to the manufacturers’ recommendations. The stained cells were analyzed with the FACS Calibur machine and Cell Quest software (BD Biosciences).

### Soft agar colony formation assay

Cells were seeded at a density of 1000 cells per well in six well plates and allowed to grow for 10 days. Clones were fixed by 4% methanol and dyewith Giemsa (Sigma Aldrich) and clone numbers were counted microscopically. The colony formation efficiency = (clone number / inculated cell number) × 100%.

### Sphere-forming assay

To investigate the self-renewal capacity of the sorted CD133^+^/CD24^+^ cells, single cell suspension prepared from parental cells or the tumor spheres of RCC CD133^+^/CD24^+^ cells was diluted to 1000 cells/ml. One microliter of the single cell suspension was plated in 96-well ultra-low plates containing 150 ml serum-free medium per well. Wells containing no cells or more than one cell were excluded, and those with one cell were marked and monitored daily under a microscope (Nikon Eclipse TE2000-S, Nikon, Japan) for 6 days and the colonies were counted. The self-renewal efficiency = (clone number / inculated cell number) × 100%.

### Quantitative RT-PCR

Total RNA was extracted from cells using TRIzol Reagent (Invitrogen, Carlsbad, CA, USA) according to the manufacturer’s instructions, and then the RNA was reverse transcribed using the PrimeScript RT Master Mix Perfect Real Time kit (TaKaRa, Dalian, China) to obtain the cDNA. Using the cDNA as the template, a real-time PCR assay was performed using the pairs of primers listed in Table 1. The 20 μL real-time PCR reaction included 0.5 μL of cDNA template, 0.25 μL of Primer F, 0.25 μL of Primer R, 10 μL of RNase-free dH_2_O, and 8 μL of 2.5× RealMasterMix (SYBR Green I). The reaction conditions included a pre-denaturation step at 95 °C for 10 s, and 40 cycles of 95 °C for 15 s and 60 °C for 60 s. After the reaction, the data were subjected to statistical analysis.

**Table 1:** Primer sequences of genes

Gene	Primer sequence
CTR2	F: 5′- TCCAGGTAGTCATCAGCT -3′
	R: 5′- TGGCAGTGCTCTGTGATGTC -3′
BCL-2	F: 5′- CTCCTGACGCTAAGAGCTTCG -3'
	R: 5′- CCAGGCTGGAAGGGAAAGAC -3′
MDR1	F: 5′- GGAAGACATGACCAGGTATGC -3′
	R: 5′- GCACATCAAACCAGCCTATCTC -3′
OCT-4	F: 5′- TTCAGCCAAACGACCATCT -3′
	R: 5′- GCTTTGCATATCTCCTGAAGA -3′
KLF4	F: 5′- CCCACATTAATGAGGCAGC -3′
	R: 5′- AGTCGCTTCATGTGGGAGAG -3′
Notch1	F: 5′- CCCGCCAGAGTGGACAGGTCAGTA -3′
	R: 5′- TGTCGCAGTTGGAGCCCTCGTTA -3′
Notch2	F: 5′- CCCACAATGGACAGGACA -3′
	R: 5′- GAGGCGAAGGCACAATCA -3′
Notch3	F: 5′- TCTCAGACTGGTCCGAATCCAC -3′
	R: 5′- CCAAGATCTAAGAACTGACGAGCG -3′
Jagged1	F: 5′- GACACCGTTCAACCTGACAGTATTA -3′
	R: 5′- GTCACAGGCATAGTGTCCAAAGA -3′
Jagged2	F: 5′- TCGGGCAGGAACTGTGAGAAGGC -3′
	R: 5′- AATCACAGTAATAGCCGCCAATCAGGT -3′
DLL1	F: 5′- AGGGGTGGAGAAGCATCTGAAA -3′
	R: 5′- AACCTGCTCGGTCTGAACTCG -3′
DLL3	F: 5′- ACGCCTGGCCTGGCACCTT -3′
	R: 5′- CCCTCTAGGCATCGGCATTCACC -3′
DLL4	F: 5′- ACAGTGAAAAGCCAGAGTGTCGG -3′
	R: 5′- TGAGCAGGGATGTCCAGGTAGG -3′
β-actin	F: 5′- AGGGGCCGGACTCGTCATACT -3′
	R: 5′- GGCGGCACCACCATGTACCCT -3′
Vimentin	F: 5′- CTTCCGCGCCTACGCCA -3′
	R: 5′- GCCCAGGCGAGGTACTCC -3′
Hey1	F: 5′- CATACGGCAGGAGGGAAAG -3′
	R: 5′- GCATCTAGTCCTTCAATGATGCT -3′
Hes1	F: 5′- AGTGAAGCACCTCCGGAAC -3′
	R: 5′- CGTTCATGCACTCGCTGA -3′

### Invasion, migration and chemotaxis assays

Cells (1 × 10^6^) incubated in serum-free medium were added on the top of the transwell chamber coated with fresh Matrigel (Gibco). The medium supplemented with 10% FBS was added to the bottom of the transwell chamber. After incubation for 48 h, the cells on top of the chamber were scraped off using a cotton swab. And the cells in the bottom of the chamber were fixed and stained with crystal violet and photographed. The crystal violet was then eluted and the eluent of each group was measured by a microplate reader to determine the optical density at 570 nm (OD570).

Migration experiments were performed in polycarbonate transwell inserts (8 μm pores, Corning Costar Corp). Cells (1 × 10^6^) in 200 μl culture medium were seeded in the upper chamber and cultured at 37 °C for 6 h. Migrating cells were fixed, stained and detected as invasion assay. For investigation of chemotaxis, SDF-1α (100 ng/ml, PeproTech) as inducer was added in the lower chamber, the other procedures were carried out as migration assay.

### Cell viability assay

The parental cells, CD133^+^/CD24^+^ sorted cells or CD133^+^/CD24^+^ sorted cells transfected with its endogenous inhibitor Numb vector pCMV6-AC-GFP-Numb (ORIGENE) or notch 1 NICD overexpression VectorpCMV6-AC-GFP-Notch1 (ORIGENE) using lipofectin2000 according to the manufacturer’s instructions, were treated with cisplatin (0, 5, 10, 15, 20 μM), sorafenib (1, 2, 3 μM), the notch pathway general pharmacological inhibitor MRK-003, or CXCR4 inhibitor AMD3100 (5 μM). Cells treated with the indicated reagents or samples in exponential growth were plated at a final concentration of 2 × 10^3^ cells per well in 96-well plates. The viability of cells was evaluated by MTT assay. The resistance to cisplatin and sorafenib was determined after treatment for 24 h. The optical density at 570 nm (OD570) of each well was measured with an ELISA reader (ELX-800 type, BioTek).

### Western blot

Cells were lysed in cell lysate, and then centrifuged at 12,000 × g for 20 min at 4 °C. The supernatant was collected and denatured. Proteins were separated in 10% SDS-PAGE and blotted onto polyvinylidene difluoride membrane (PVDF). The PVDF membrane was treated with TBST containing 50 g/L skimmed milk at room temperature for 4 h, followed by incubation with the primary antibodies: anti-CTR2 (1:200, Novusbio), anti-BCL-2 (1:500, Immunoway), anti-OCT-4 (1:1000, Proteintech), anti-KLF4 (1:500, Proteintech), anti-MDR1 (1:200, Santa),, anti-Notch1 ICD (1:1000, Abcam), anti-Notch2 ICD (1:1000, Abcam), anti-Jagged1 (1:500, Abcam), anti-Jagged2 (1:1000, Abcam), anti-DLL1 (1:500, Abcam), anti-DLL4 (1:500, Abcam), anti-Notch ICD (1:1000, Cell Signaling), anti-SDF-1 (1:1000, Abcam), anti-CXCR4 (1:2000, Abcam) and anti-β-actin (1:1000, Cell signaling) respectively, at 37 °C for 1 h. Membranes were rinsed and incubated for 1 h with the correspondent peroxidase-conjugated secondary antibodies. Chemiluminent detection was performed with the ECL kit (Pierce Chemical, Rockford, IL, USA). The amount of the protein of interest, expressed as arbitrary densitometric units, was normalized to the densitometric units of ß-actin.

### Tumorigenicity assay

Animal experiments were performed in strict accordance with the Guide for the Care and Use of Laboratory Animals of Hunan Provincial People’s Hospital. The protocol was approved by the Committee on the Ethics of Animal Experiments of Hunan Provincial People’s Hospital. NOD/SCID mice at age of 3–5 weeks, male, were maintained in pathogen-free conditions at animal facility. The Cells pretreated with MRK-003 or Numb were resuspended in serum-free medium and mixed with Matrigel at the ratio of 1:1. NOD/SCID mice were randomly divided into 4 groups (*n* = 6 per group). Indicated cells of 3 dosages (1 × 10^5^, 1 × 10^4^, and 1 × 10^3^) were inoculated subcutaneously into the inguinal folds of NOD/SCID mice. Tumor formation was evaluated regularly after injection by palpation of injection sites. Tumor volume was calculated using the equation (Length × Weight^2^)/2. At the end of experiment, the mice were sacrificed under deep anesthesia with pentobarbital. The tumors were then dissected and captured.

### Immunocytochemistry analysis

The tumor tissue was fixed in 4% paraformaldehyde overnight. Tissue specimens were then cut at 5 μm thickness and a standard immunostaining procedure was performed using antibodies against actived-capase-3 p17 (1: 100, Bioworld Technology, Inc.) and PCNA (1: 50, ABZOOM). The mean optical density value (D) and area (A) of brown particles in three visual fields of each section were calculated by the Leica Q550 image analysis system (Leica, German). The expression levels of target molecules in tissues were evaluated using the formula: integral density = D × A.

### Statistical analysis

All data are presented as mean ± standard deviation. The means of groups were compared with one-way analysis of variance, and after checking for equal variance, comparisons between two means were performed using the least significant difference (LSD) method. Student’s *t*-test was used for two group’s comparison. Analysis of variance was used for clinical statistical analyses. In all cases, *P* < 0.05 was considered with statistical significant.

## Results

### Isolation of CD133^+^/CD24^+^ cells and characterization of its stemness markers

**Fig. 1:**
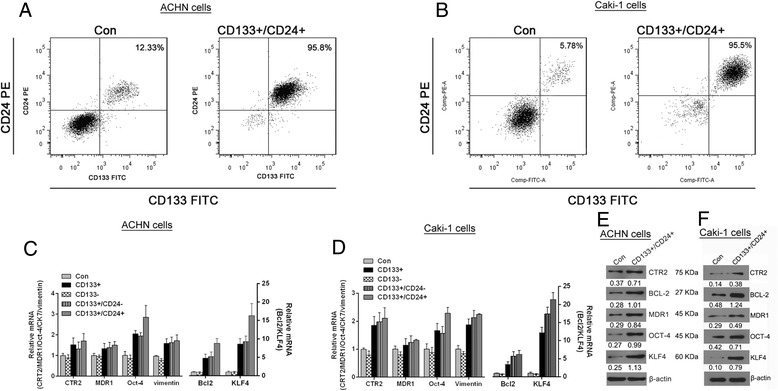
Identification of the purity of sorting CD133^+^/CD24^+^ cells and its *stemness markers*. The purity of sorting CD133^+^/CD24^+^ACHN **a** or Caki-1 cells **b** were detected by flow cytometry analysis. The relative mRNAs (**c** and **d**) and protein (**e** and **f**) expressions of *stemness markers* as indicated were determined by RT-PCR. Control (Con):parental ACHN or Caki-1 cells

To establish of RCC CSCs models from renal carcinomas cells, the ACHN and Caki-1 cell line cells were subjected to immunomagnetic bead separation and the purity of CD133^+^/CD24^+^ cells was detected by flow cytometry. As shown in Fig. 1a and b, the purity of CD133^+^/CD24^+^cells sorted from ACHN and Caki-1 cell lines by immunomagnetic bead separation reached up to 95.8 and 95.5%, respectively, much higher than that in control parental cells. Then the relative mRNAs (Fig. 1c and d) and protein (Fig. 1e and f) expression of CTR2, BCL-2, MDR1, OCT-4, KLF4,Vimentin and in CD133^+^/CD24^+^ cells were determined by qRT-PCR and western blot analysis, respectively. The results confirmed that increased CTR2, BCL-2, MDR1, OCT-4, KLF4, Vimentin and decreased expression were detected in theCD133^+^, CD133^+^/CD24^−^ and CD133^+^/CD24^+^cells of both cell lines, compared to responding parental cells or CD133^−^cells. And the expression of Oct-4, Bcl-2 and KLF4 in CD133^+^/CD24^+^cells of both cell lines was higher than that in CD133^+^/CD24^−^ cells . It implies that the enrichment of CD133^+^/CD24^+^cellsfrom ACHN or Caki-1 cell lines may be beneficial in establishment of the RCC CSCs models under sphere-forming culture.

### CD133^+^/CD24^+^ cells have functional features of CSCs

**Fig. 2:**
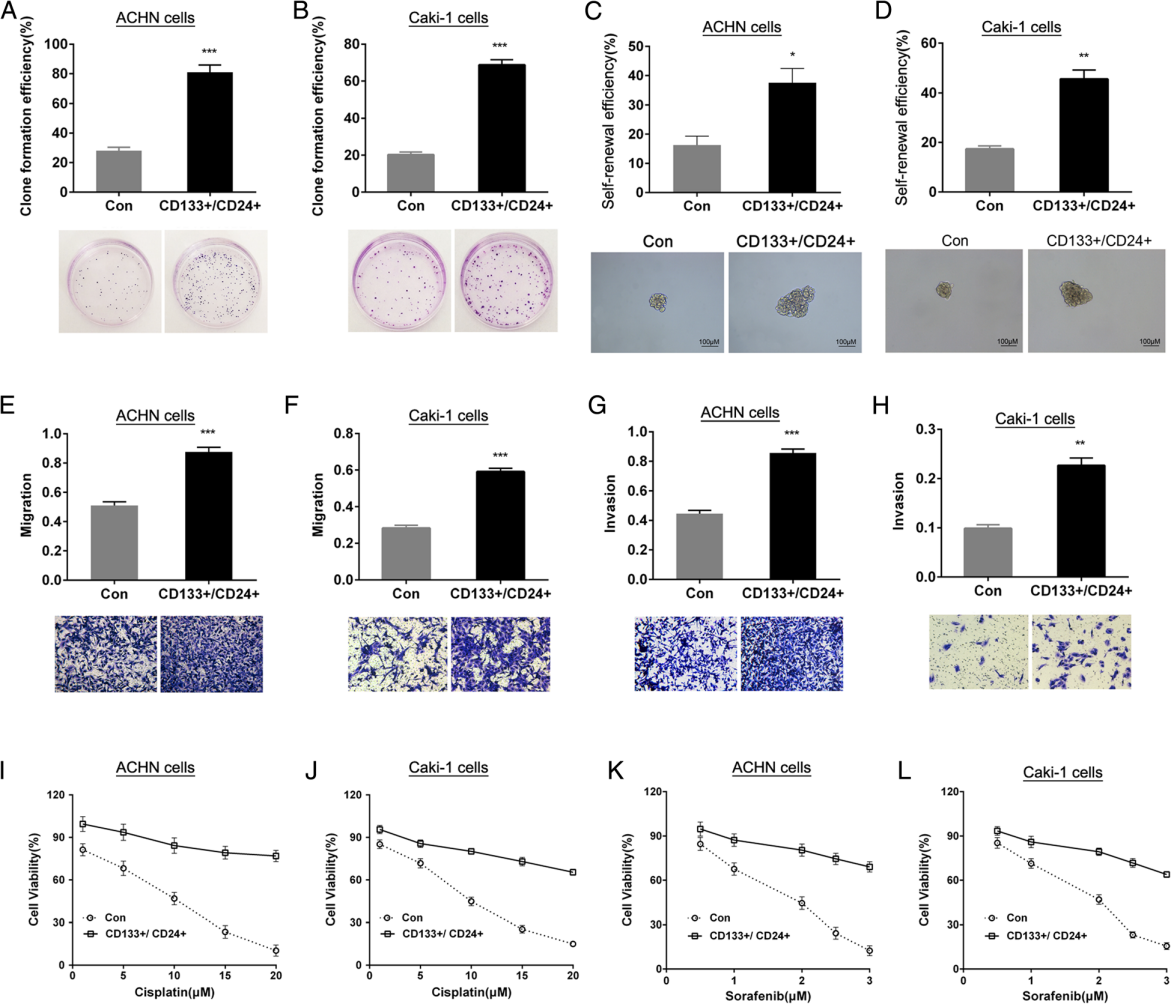
Identification of stem-like features of CD133^+^/CD24^+^ cells. The clone formation efficiency of CD133^+^/CD24^+^ACHN **a** and Caki-1 **b** cells was determined in soft agar. The self-renewal efficiency of CD133^+^/CD24^+^ACHN **c** and Caki-1 **d** cells was detected by sphere formation assay. The migratory (**e** and **f**) and invasive (**g** and **h**) capability of CD133^+^/CD24^+^ cells were detected by transwell assay. The sensitivity of CD133^+^/CD24^+^cells to cisplatin (**i** and **j**) and sorafenib (**k** and **l**) was determined by MTT. ^*^ *P* < 0.05 VS. control; ^**^ *P* < 0.01 VS. control;^***^ *P* < 0.001 VS. control; Control (Con):parental ACHN or Caki-1 cells)

To validate whether the CD133^+^/CD24^+^ cells derived from ACHN or Caki-1 cell lines have stem cell behavior, the soft agar colony formation assay, sphere-forming assay, invasion and migration by transwell assay, drug sensitivity by MTT assay and tumorigenicity assay in vivo were performed. The results showed that, compared to renal carcinomas ACHN or Caki-1 parental cells (control, Con), the CD133^+^/CD24^+^ cells of both cell lines have higher clone formation efficiency in soft agar medium (Fig. 2a and b), suggesting the CD133^+^/CD24^+^ cells have growth features of stem cells; single cells sphere-forming assay results showed that the CD133^+^/CD24^+^ cells could form a greater number and bigger size of non-adherent spheres which is called renal carcinomas sphere-forming cells (SFCs), indicating that the CD133^+^/CD24^+^ cells have stronger self-renewal capability (Fig. 2c and d); the transwell data confirmed that the CD133^+^/CD24^+^ cells possessed enhanced migratory and invasive capability (Fig. 2e–h); cisplatin (0, 5, 10, 15, 20 μM) and sorafenib (1, 2, 3 μM) inhibited the proliferation of parental cells in a dose-dependent manner, but the cell viability in CD133^+^/CD24^+^ cells was significantly higher than that in parental cells (Fig. 2i–l), suggesting that the CD133^+^/CD24^+^cells have resistance to cisplatin and sorafenib; moreover, the results of tumorigenicity in vivo showed that 1 × 10^4^ of CD133^+^/CD24^+^ cellscultured in stem cell conditioned medium were sufficient to induce tumor in NOD/SCID mice, however, the ACHN or Caki-1 cells cultured in the uniform medium needed at least 1 × 10^5^cells. Under the condition of the uniform inoculum size, the tumor incidence in vivo induced by CD133^+^/CD24^+^ cells was higher than that in the parental cells (Table 2). The above data demonstrate that the CD133^+^/CD24^+^ cells sorted from ACHN or Caki-1 cell lines and maintained in stem cell conditioned medium have the clear functional features of CSCs and thus can be used as RCC CSCs models for the followed study.

**Table 2:** Xenotransplantation of cells into NOD/SCID mice

Cells + Treatment	Inoculum size	Tumor incidence		Tumor volume (mm^3^)	
		ACHN cell line	Caki-1 cell line	ACHN cell line	Caki-1 cell line
Parental cells	1 × 10^5^	6/6	6/6		
	1 × 10^4^	4/6	5/6	501.17 ± 46.31	544.67 ± 62.26
	1 × 10^3^	1/6	1/6		
CSCs	1 × 10^5^	6/6	6/6		
	1 × 10^4^	6/6	6/6	774.67 ± 54.04	756.67 ± 70.32
	1 × 10^3^	3/6	3/6		
CSCs + MRK003	1 × 10^5^	5/6	5/6		
	1 × 10^4^	2/6	3/6	306.17 ± 56.56	378.50 ± 38.34
	1 × 10^3^	0/6	1/6		
CSCs + Numb	1 × 10^5^	5/6	5/6		
	1 × 10^4^	3/6	3/6	317.17 ± 82.34	377.50 ± 51.93
	1 × 10^3^	0/6	1/6		

### Notch pathway is up-regulated in RCC CSCs

**Fig. 3:**
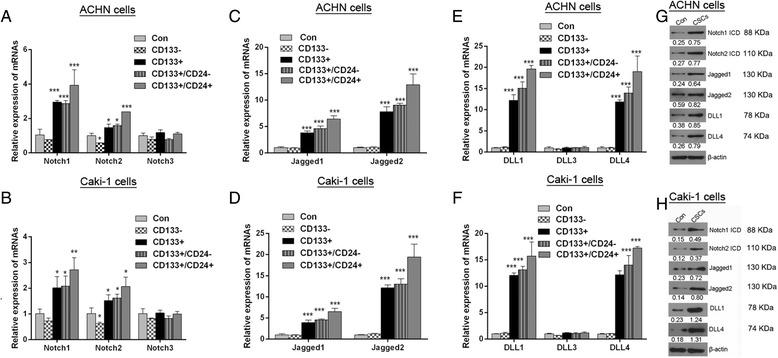
Identification of the expression of Notch pathway in RCC CSCs. The relative mRNA levels of notch1-3 (**a** and **b**), Jagged1/2 (**c** and **d**), DLL1, DLL3 and DLL4 (**e** and **f**) were detected using RT-PCR. (**g** and **h**) The up-regulation of notch1/2, Jagged1/2, DLL1/4 protein was validated by western blot analysis. ^*^ *P* < 0.05 VS. control; ^**^ *P* < 0.01 VS. control;^***^ *P* < 0.001 VS. control; Control (Con):parental ACHN or Caki-1 cells

Notch pathways play an important role in regulation of functions and features of stem cells. Here, we found that Notch1 and Notch2 mRNA levels in RCC CSCs (CD133^+^/CD24^+^) were significantly higher than that in parental cells, however, Notch3 mRNA levels in the both types of cells were not different in statistical significance (Fig. 3a and b). Then it was discovered that the mRNAs levels of their corresponding ligandsJagged1 and Jagged2 in RCC CSCs were also markedly elevated, compared to its expression in parental cells (Fig. 3c and d). The expression of DLL1, DLL3 and DLL4 in the both types of cells were compared and the results showed that only DLL1 and DLL4 were significantly increased in RCC CSCs without alternation of DLL3 in statistical significance (Fig. 3e and f). Western blot analysis further confirmed that Notch1, Notch2, Jagged1, Jagged2, DLL1 and DLL4 protein levels were also enhanced in RCC CSCs (Fig. 3g and h). Those results demonstrate that the notch pathways in RCC CSCs derived from ACHN or Caki-1 cell lines are abnormal activated.

### RCC CSCs maintaining stemness depends upon notch1/2

**Fig. 4:**
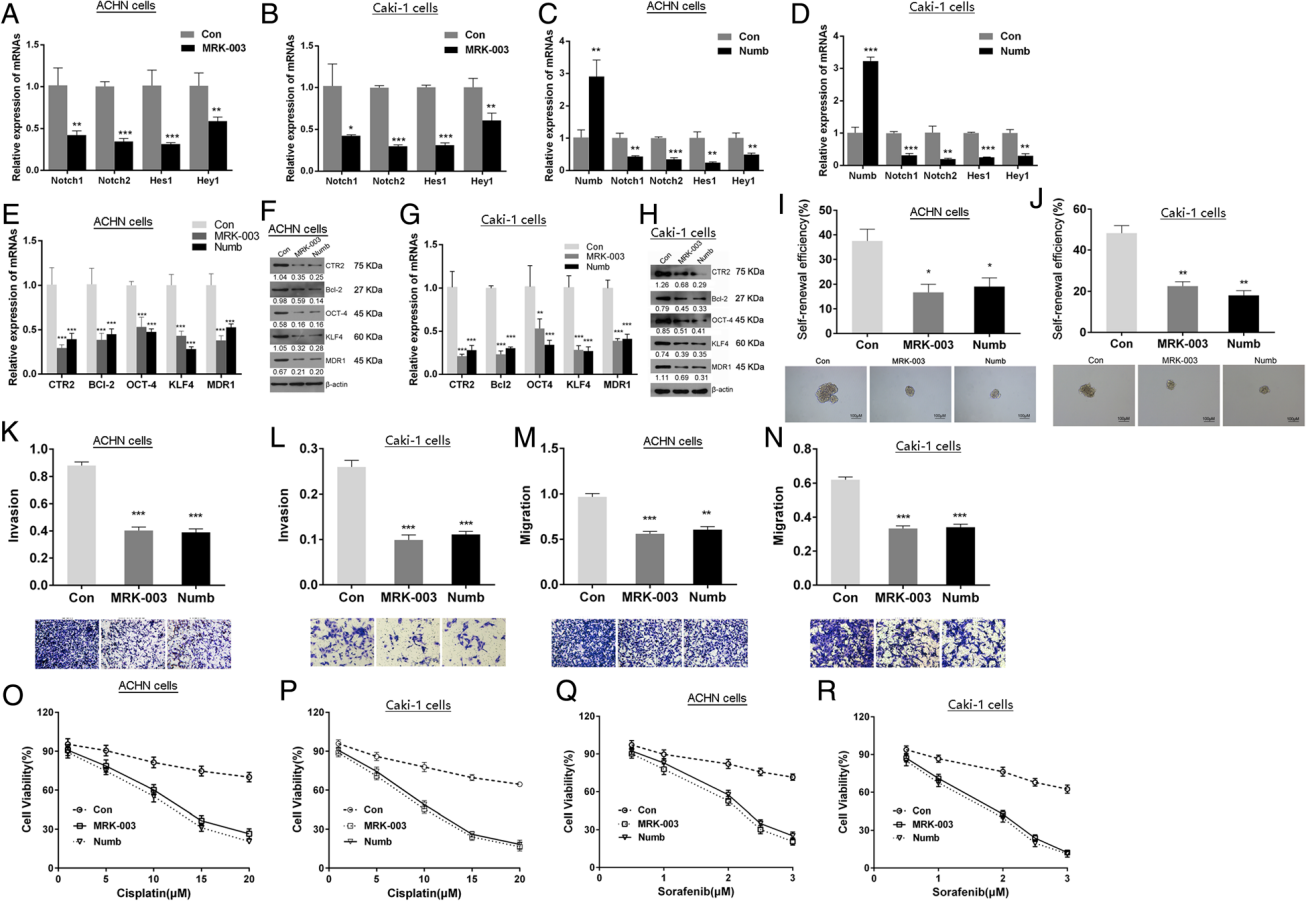
The effects of notch signaling on stemness of RCC CSCs. The pharmacological (MRK-003) and endogenous (*Numb*) notch inhibitor suppressed the expression of notch1, notch2, Hes1 and Hey1 mRNAs (**a**, **b**, **c** and **d**), and stemness markers mRNAs (**e** and **g**) and proteins (**f** and **h**), *decreased* the self-renewal efficiency (**i** and **j**), invasion (**k** and **l**), migration (**m** and **n**), and sensitivity to cisplatin (**o** and **p**) and sorafenib (**q** and **r**) in RCC CSCs. ^*^ *P* < 0.05 VS. control; ^**^ *P* < 0.01 VS. control;^***^ *P* < 0.001 VS. control; Control (Con): the RCC CSCs treated without inhibitor)

**Fig. 5:**
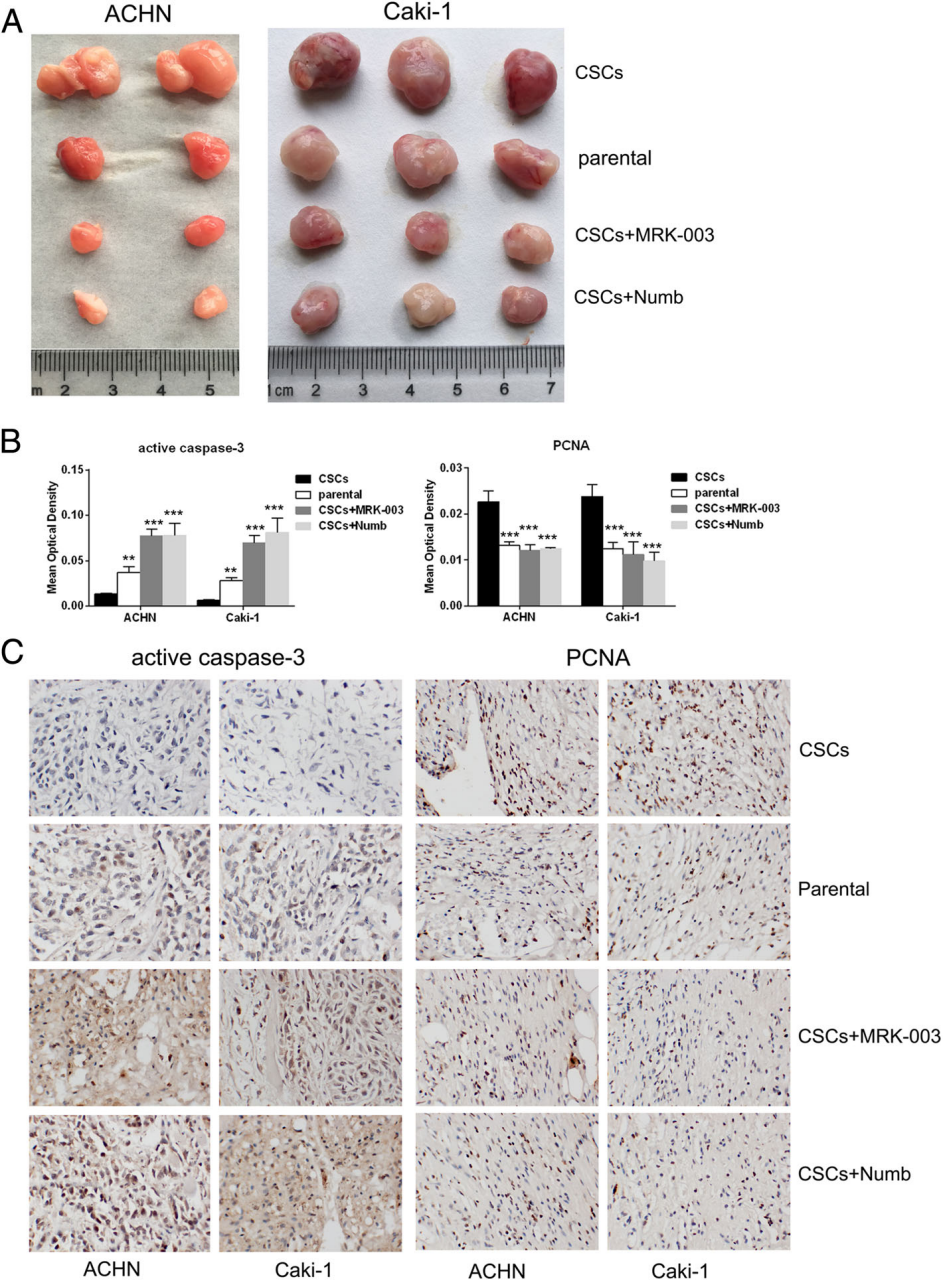
The effects of notch signaling on proliferation of RCC CSCs in vivo. **a** The pharmacological (MRK-003) and endogenous (*Numb*) notch inhibitor suppressed the growth of RCC CSCs in NOD/SCID mice. (**b** and **d**) Immunohistochemistry analysis showed that *increased* actived-caspase-3 and *decreased* PCNA were detected in tumor tissue from RCC CSCst reated with RAK-003 or transfected with Numb vector.^*^ *P* < 0.05 VS. control; ^**^ *P* < 0.01 VS. control;^***^ *P* < 0.001 VS. control; Control (Con): the RCC CSCs treated without inhibitor)

To validate whether notch1 and notch2 play functional role in regulation of stemness of RCC CSCs, the general pharmacological inhibitor MRK-003 and its endogenous inhibitor Numb (ORIGENE) of notch pathway were applied. As shown in Fig. 4a and b, MRK-003 significantly suppressed the expression of notch1, notch2, Hes1 and Hey1 in RCC CSCs. And transfection of Numb vectors markedly increased Numb expression and significantly inhibited notch1, notch2, Hes1 and Hey1 in RCC CSCs (Fig. 4c and d). Inhibition of notch1/2 by MRK-003 or Numb resulted in down-regulation of the stemness markers CTR2, BCL-2, OCT-4, KLF4 and MDR1 (Fig. 4e–h), reduced self-renewal (Fig. 4i and j), invasive and migration capability (Fig. 4k–n), enhanced sensitivity to cisplatin and sorafenib (Fig. 4o–r), and decreased tumorigenicity (Fig. 5a and Table 2) in RCC CSCs. Furthermore, the immunehistological analysis showed that the elevated active caspase3 and reduced PCNA were detected in the xenografts from RCC CSCs treated with MRK-003 or transfected with Numb (Fig. 5b and c), indicating inhibition of notch1/2 increased apoptosis and decreased proliferation of RCC CSCs in vivo. It confirms that maintenance stemness of RCC CSCs, at least, partly depends upon the activation of notch1 and notch2.

### Notch1 contributes to chemotaxis of RCC CSCs by CXCR4/SDF-1 axis

**Fig. 6:**
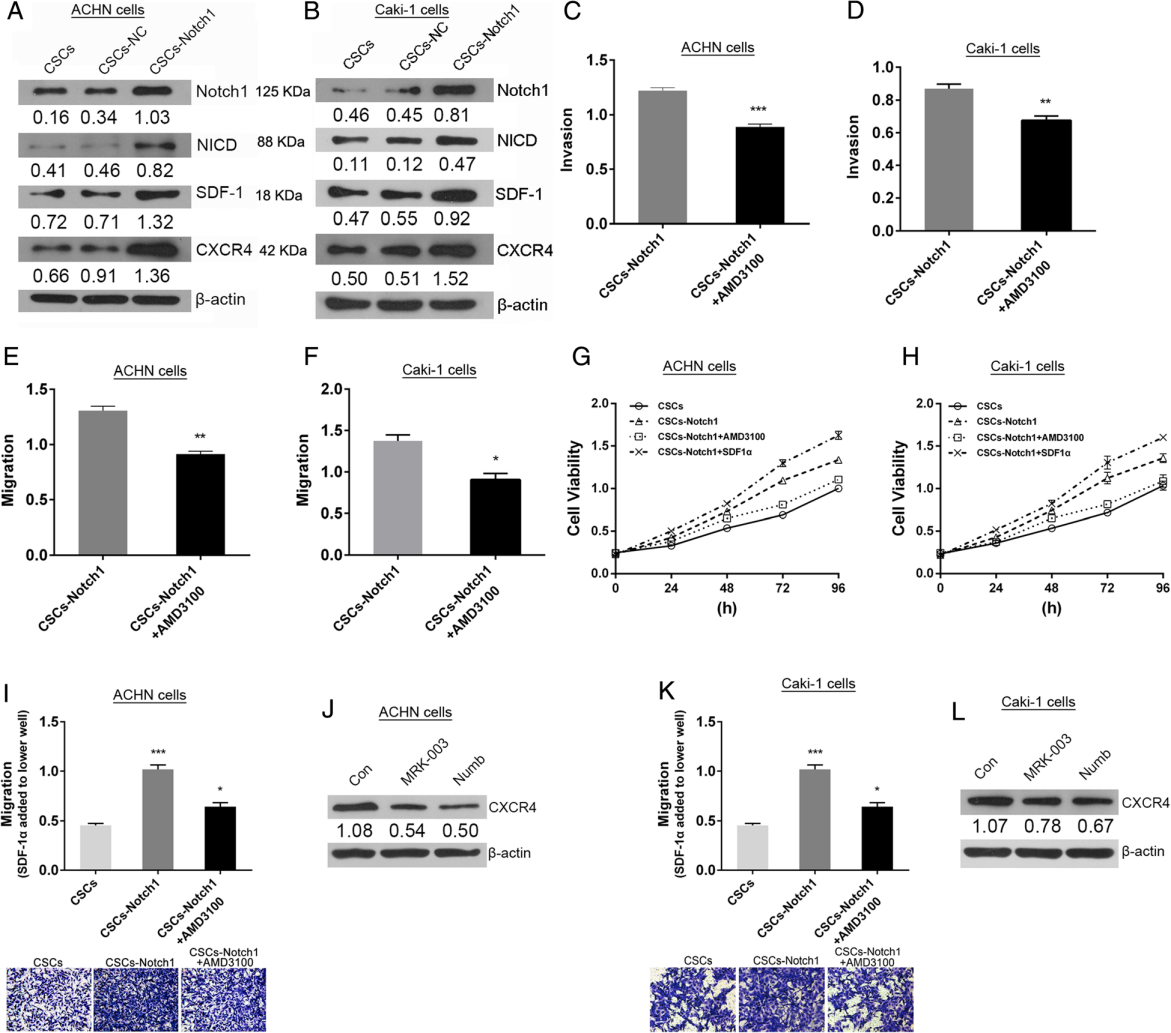
The effects of overexpression of notch 1 on chemotaxis of RCC CSCs induced by SDF-1. (**a** and **b**) Increased SDF-1 and CXCR4 induced by overexpression of notch1 in RCC CSCs. CXCR4 inhibitor AMD3100 decreased invasion (**c** and **d**) and migration (**e** and **f**) of RCC CSCs overexpressed by notch1. (**g** and **h**) Addition of SDF-1α and AMD3100 affected the cell viability of RCC CSCs overexpressed by notch1. (**i** and **k**) Addition of SDF-1α in the lower well in transwell assay, AMD3100 reduced overexpression of notch1-mediated chemotaxis in RCC CSCs. (**j** and **l**) Suppression of notch 1 induced by its inhibitor decreased CXCR4 in RCC CSCs. ^*^ *P* < 0.05 VS. indicated group; ^**^ *P* < 0.01 VS. indicated group;^***^ *P* < 0.001 VS. indicated group

To investigate the mechanisms underlying notch regulation of chemotaxis of RCC CSCs, the notch1 overexpression RCC CSCs model (CSCs-Notch1) were successfully constructed and western blot analysis showed that overexpression of notch1 induced up-regulation of CXCR4 and SDF-1 (Fig. 6a and b). Treatment of RCC CSCs overexpressing notch1 with CXCR4 inhibitor AMD3100 (5 μM, 24 h) could suppress its invasive and migratory capability (Fig. 6c–f). It suggests that notch1 contributes to invasion and migration of RCC CSCs via up-regulation of CXCR4. As shown in Fig. 6g and h, overexpression of notch1 increased the cell viability of RCC CSCs. And addition of CXCR4 inhibitor partly rescued overexpression of notch1 mediated cell viability enhancement. But addition of recombination protein SDF-1α could further boost overexpression of notch1 mediated enhancement of cell viability. Those results indicate that notch1 promotion of proliferation of RCC CSCs is closely involved in activation of CSCR4/SDF-1 axis. To investigate the effects of notch1 on chemotaxis in RCC CSCs, SDF-1α was added in the lower well in the transwell assays. The results showed that overexpression of notch1 significantly increased the migration of ccRCC CSCs. But addition of CXCR4 inhibitor partly rescued overexpression of notch1 mediated enhancement of cell migration (Fig. 6i and k). As shown in Fig. 6j and l, the expression of CXCR4 decreased in RCC CSCs in which notch1 signaling was suppressed by its inhibitor. Those results demonstrate that notch1 increases SDF-1-induced chemotaxis of RCC CSCs via up-regulation of CXCR4.

## Discussion

CSCs have been identified inside different cancers and considered as the origin of the initiation, growth, metastasis, chemo-resistance and recurrence of malignant tumors. Clinically, currently used treatment strategies for cancers mostly target somatic tumor cells rather than CSCs. For the development of efficient therapies against CSCs, it is necessary to isolate and characterize CSCs from tumor tissues or cell lines, and reveal its functional features and stemness maintenance mechanisms. It has been revealed that CD133, CD24, CD105, Snail, Nanog, Twist, OCT-3/4, CRT2, BCL-2,MDR1, KLF4 and so on are stemness markers in CSCs of renal cell carcinoma [13, 16] or other types of tumor [23, 24]. Here, we successfully isolated and characterized the CD133^+^/CD24^+^ subpopulation of RCC ACHN and Caki-1 cell line cells using the magnetic-activated cell sorting (MACS) system and cytometry analysis. And the increased expression of stemness genes (CTR2, BCL-2, MDR1, OCT-4, KLF4, Vimentin) were discovered in CD133^+^/CD24^+^ACHN and Caki-1 cells.

CD133 expression is possibly associated with worse prognosis in tumor patients [25] and has been used as a stem cell marker in various tumors including renal cell carcinoma, however, CD133 as a single marker may not be sufficient for CSC identification in RCC [16]. Galleggiante and his colleagues [26] found that the CD133^+^/CD24^+^ tumor cells isolated from human renal cell carcinoma tissues possessed the CSCs characteristics such as self-renewal ability and multi-differentiation potential. Our results further confirmed that CD133^+^/CD24^+^ tumor cells isolated from RCC ACHN or Caki-1 cell line cells expressed by higher level of stemness marker and possessed self-renewal ability validated by soft agar colony formation and spheres formation assays, resistance to cisplatin and sorafenib, stronger ability to form tumor in vivo, and higher invasive and migratory potential validated bytranswell assay. Moreover, CD133^+^/CD24^+^ RCC ACHN cells showed stronger self-renewal ability compared to its CD133^+^ tumor cells (data not shown). It suggests that those sorting ccRCC CD133^+^/CD24^+^cells have stemness markers as well as functional properties of CSCs and were thus used as CSCs model of RCC in the subsequent functions and mechanisms investigation.

Notch pathway, comprising 4 receptors (Notch 1–4) and 5 ligands (DLL-1, DLL-3, DLL-4, Jagged-1 and Jagged-2) in adjacent cells [27], plays an important role in regulation of cellular communication in embryogenesis and stemness, differentiation and growth of stem cell [28, 29]. Notch may play a role in tumourigenesis by inhibiting differentiation, promoting survival, or accelerating proliferation. The aberrant activation of notch signaling pathway may contribute to development of some tumors including melanoma, glioma, breast carcinoma, colon carcinoma, cervical cancer and so on [30, 31]. But it is also reported that notch signaling may serve as a suppressor in a few tumors, for example, forebrain tumor subtypes [22]. Therefore, the special roles of notch pathway in development of tumor may depend upon tumor types. Studies indicate that notch signaling pathway takes part in regulation of stemness properties and functions including self-renewal, differentiation, chemosensitivity, invasion and migration in CSCs [28] derived from hepatocellular carcinoma [32], colorectal carcinoma [33], pancreatic cancer [34], esophageal adenocarcinoma [35], glioblastoma [36], etc. It is reported that notch1, notch3 and jagged1 are highly expressed in RCC and blockage of notch signaling can suppress its growth [37, 38]. And high jagged1 expression predicts poor outcome in RCC [39]. However, the expression pattern, special functions and action mechanisms of notch pathway in RCC CSCs remain elusive.

Therefore, we examined the expression of the 4 receptors and 5 ligands of notch pathway in RCC CSCs derived from ACHN and Caki-1 cells and found that Notch1, Notch2, Jagged1, Jagged2, DLL1 and DLL4 were significantly enhanced in RCC CSCs, suggesting that notch signaling pathways in RCC CSCs are aberrant activated. While notch1/2 was suppressed by its pharmacological inhibitor MRK-003 or its endogenous inhibitor Numb, the expression of stemness markers (CTR2, BCL-2, OCT-4, KLF4 and MDR1) and stemness functional properties (self-renewal, high invasion and migration, resistance to cisplatin and sorafenib and strong tumorigenicity) were inhibited in RCC CSCs. It confirms that RCC CSCs sustaining stemness, at least, partly depends upon the activation of notch1/2 possibly by Jagged1, Jagged2, DLL1 or DLL4 in adjacent CSCs, strongly supporting a crucial role of the notch pathways inRCC CSCs subset maintenance.

It is well known that BCL-2 is a member of anti-apoptotic protein family and its up-regulation will favor RCC CSCs survival via resistance to drugs or cytokines induced pro-apoptotic action. Up-regulation of drug resistance gene including MDR1 is associated with increased chemo-resistance of RCC CSCs. OCT-4 may play a critical role in CSCs maintaining self-renewal [40]. KLF4 is an important transcript factor in induction of dedifferentiation [41]. OCT-4 and KLF4 function in sustaining the pluripotent of stem cell. Blockage of notch signaling in RCC CSCs led to the down-regulation of anti-apoptotic gene, drug resistance gene and pluripotent gene and loss of its stemness characteristics and functions. It implies that the aberrant activation of notch signaling could up-regulated those stemness-associated genes are possible mechanisms underlying notch pathway serves as a crucial promoter in RCC CSCs maintenance.

The high mortality of RCC diseases correlates with its significant propensity to metastasize in an organ specific manner. Enhanced CXCR4 expression was detected in several human renal carcinoma samples, while only minimal CXCR4 expression was detected in normal kidney tissues [42]. It has been suggested that CXCR4 expression by tumor cells, plays a critical role in cell metastasis by a chemotactic gradient to organs expressing the ligand SDF-1. SDF-1 as the receptor of CXCR4 promotes CXCR4-expressing RCC cells metastasis to specific organ expressing SDF-1 [43]. Moreover, our data further demonstrate that notch1 promotes SDF-1-induced chemotaxis of RCC CSCs via up-regulation of CXCR4. Our findings are consistent with the previous reports that CXCR4 functions in maintenance of renal cell carcinoma-initiating cells derived from renal carcinoma cell line RCC-26 an RCC-53 [44] and its expression in mesenchymal stem cells is regulated by Notch signaling [45]. Thus, it is suggested that aberrant activation of notch1 signaling may enhance invasive and migratory ability of RCC CSCs that may promote its metastasis to special origin. Our results also showed that overexpression of notch1 resulted in increased SDF-1 in RCC CSCs. Whether this implies SDF-1/CXCR4 axis play a functional role in itself homing and proliferation in microenviroment need further study.

Taken together, our findings demonstrate the crucial role of notch pathway in RCC CSCs maintenance and provide potent preclinical evidence supporting notch signaling pathway as an optional target for eliminating the CSCs in RCC tissues. Thus, it is a promising therapeutic option to combine blockage of notch signaling pathway and the standard chemotherapy mainly targeting bulk tumor. This strategy may offer the hope to partly resolve the problems such as metastasis, recurrence and chemoresistance in clinical treatment of RCC and greatly improve the quality of life in patients with RCC.

## Conclusions

The research confirmed that notch1, notch2, Jagged1, Jagged2, DLL1 and DLL4 were over-expressed in RCC CSCs, and blockage of Notch1 or notch2 using pharmacological inhibitor MRK-003 or its endogenous inhibitor Numb resulted in partial loss of its stemness features: self-renewal, chemoresistance, invasive and migratory potential, and tumorigenesis in vivo. Moreover, it is confirmed that notch signaling promotes the chemotaxis of RCC CSCs by SDF-1/CXCR4 axis. Our results provide a new mechanism of RCC CSCs maintaining stemness via notch pathway as well as a potential therapeutic target in human RCC.
